# Big data in the healthcare system: a synergy with artificial intelligence and blockchain technology

**DOI:** 10.1515/jib-2020-0035

**Published:** 2021-08-18

**Authors:** Reyes-González Juan Pablo, Díaz-Peregrino Roberto, Soto-Ulloa Victor, Galvan-Remigio Isabel, Castillo Paul, Ogando-Rivas Elizabeth

**Affiliations:** Department of Radiology, Hospital Angeles del Pedregal, Mexico City, Mexico; Department of Technology Innovation, hdm.world, Florida, USA; Institute of Biology, Otto von Guericke University, Magdeburg, Germany; Emergency Department, Hospital General #48, Instituto Mexicano del Seguro Social, Mexico City, México; College of Medicine, Universidad Nacional Autonoma de Mexico, Mexico City, Mexico; Division of Pediatric Hematology Oncology, Department of Pediatrics, University of Florida, Gainesville, FL, USA; Department of Neurosurgery, Brain Tumor Immunotherapy Program, McKnight Brain Institute, University of Florida, Gainesville, FL, USA

**Keywords:** artificial intelligence, big data, blockchain, healthcare system, machine learning

## Abstract

In the last decades big data has facilitating and improving our daily duties in the medical research and clinical fields; the strategy to get to this point is understanding how to organize and analyze the data in order to accomplish the final goal that is improving healthcare system, in terms of cost and benefits, quality of life and outcome patient. The main objective of this review is to illustrate the state-of-art of big data in healthcare, its features and architecture. We also would like to demonstrate the different application and principal mechanisms of big data in the latest technologies known as blockchain and artificial intelligence, recognizing their benefits and limitations. Perhaps, medical education and digital anatomy are unexplored fields that might be profitable to investigate as we are proposing. The healthcare system can be revolutionized using these different technologies. Thus, we are explaining the basis of these systems focused to the medical arena in order to encourage medical doctors, nurses, biotechnologies and other healthcare professions to be involved and create a more efficient and efficacy system.

## 1 Introduction

In the past decades, big data was based on the length and complexity of the information, essentially catalogued as information that cannot be stored nor analyzed by traditional means due to its enormous dimensions and convolutions [1], but later this information started to be stored, managed and analyzed through different technologies [2], that helped us to translate that information in a value resource to create, innovate and improve our daily activities.

However, there was not an official definition of Big data, one of the accurate definitions was made by De Mauro et al. [3] by means of a scientific consensus. Therefore, Big Data was then defined as “the representation of information assets characterized by a high volume, velocity and variety to require specific technology and analytical methods for its transformation into value”.

**Figure 1:**
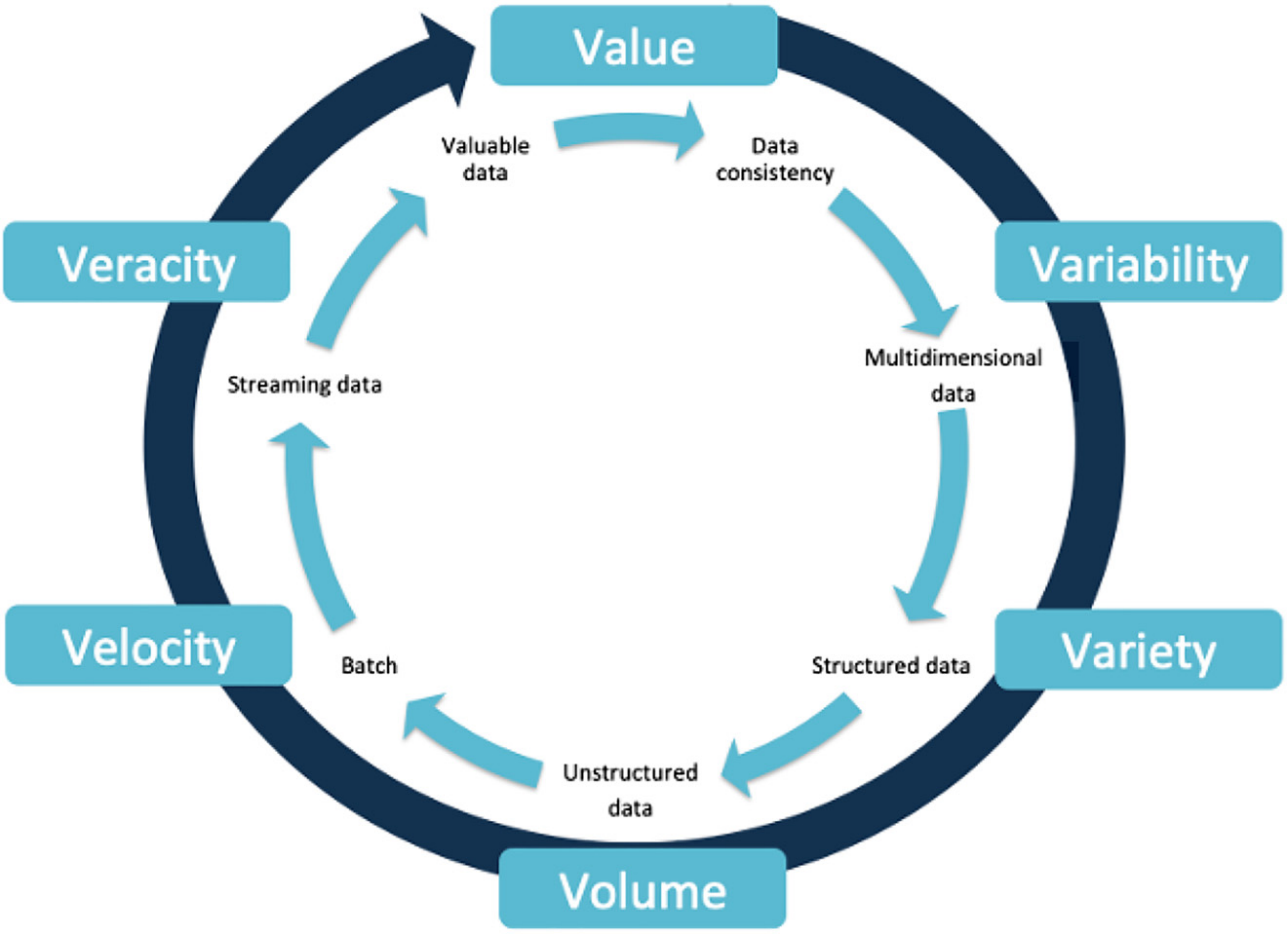
Features of big data. The cardinal features of big data must be built based on the value, variability, variety, volume, velocity and veracity, in order to achieve those features, the inner circle shows the process that data has to follow.

The backbone of big data is made of the six “V” (Figure 1). In last decades, there has been growing potential usefulness of massive quantities of data, and its use, in transforming personal care, clinical care and health using artificial intelligence, blockchain and machine learning, with this context, the purpose of the present work is to review the usefulness of big data in the context of healthcare applications with the aim of encouraging the reader in the development and promotion of these technologies in the healthcare field, an issue that today is a reality and that encourages the use of multiple technologies for the search (Table 1).

**Table 1:** European Commission supported initiatives concerning activities that involve the use of Big Data, Machine learning, Blockchain and artificial intelligence in health in Europe from 2019 to 2020.

Acronym	Title	Start date	End date
AI-MICADIS	Artificial Intelligence System for Multi-Cancer Detection Support	2019-01-01	2019-06-30
MAMMO1	Deep learning for mammography: Improving accuracy and productivity in breast cancer diagnosis	2019-01-01	2019-04-30
FETFX	Stimulating effects of Future and Emerging Technologies through communication and outreach	2019-01-01	2021-06-30
SPHINX	A Universal Cyber Security Toolkit for Health-Care Industry	2019-01-01	2021-12-31
SmartEater	Enhancing recovery from eating and weight disorders using mHealth and psychological theory	2019-01-01	2020-06-30
MASTER	Microbiome Applications for Sustainable food systems through Technologies and EnteRprise	2019-01-02	2023-01-01
Brain Health Toolbox	The Brain Health Toolbox: Facilitating personalized decision-making for effective dementia prevention	2019-02-01	2024-01-31
Magentiq Eye	Automatic Polyp Detection System	2019-02-01	2019-07-31
LifeTime	Revolutionizing Healthcare by Tracking and Understanding Human Cells during Disease	2019-03-01	2020-06-30
VagX	Diagnostic tool for Vaginitis	2019-05-01	2019-10-31
DIGI-B-CUBE	Digital Enterprise Innovations for Bioimaging, Biosensing and Biobanking Industries	2019-05-01	2022-04-30
DIABETE SMART	The first Clinically Validated Al-powered Diabetes Assistant	2019-05-01	2019-08-31
BrainPatch	BrainPatch - Breakthrough non-invasive brain stimulation using Al	2019-06-01	2019-11-30
PERISCOPE	Perioperative infection prediction	2019-06-01	2019-11-30
EggSorter	Device and method for automatized egg cell inspection and sorting	2019-06-01	2020-02-29
HAPGuide	Smart and sensing robotic system for endovascular interventions with haptic feedback	2019-07-01	2019-12-31
Cortx	Intelligent debt recovery platform	2019-07-01	2019-12-31
SCMS	ScreenCancer Mole Scanner - Easily accessible mole scanning service for early detection of skin cancer	2019-08-01	2019-11-30
HYGGii	The therapist in your pocket - Al assisted ecosystem for enabling a patient-centric approach to mental health	2019-08-01	2019-11-30
SIPAR	A revolutionary gait analysis system for smart injury and performance management	2019-10-01	2020-03-31
PLS	Perinatal Life Support System: Integration of Enabling Technologies for Clinical Translation	2019-10-01	2024-09-30
APRICOT	Anatomically Precise Revolutionary Implant for bone Conserving Osteoarthritis Treatment	2019-10-01	2023-09-30
EDAS HEALTHCARE	Al-based infectious diseases diagnosis in seconds	2019-11-01	2020-02-29
Miiskin	ML-powered app and platform to identify skin changes over time	2019-12-01	2020-03-31
aiVision	Innovative Al-based Ophthalmologic Diagnosis	2019-12-01	2020-03-31
MiChomAbs	Synthetic Mini-Chromosomes for Antibody Production	2019-12-01	2020-05-31
ADAM 0 Robot	The future of precise personalised robotic physiotherapy	2019-12-01	2020-03-31
NIGHTWATCH	NightWatch - Clinically-proven detection and alert system for critical nocturnal epileptic seizures	2019-12-01	2020-03-31
EVIE 2.0	A slow release insemination that doubles the success rate of the most common first line treatment of infertility	2019-12-01	2020-02-29
iScan	Automated retinal scans for early detection of diabetic eye diseases through use of artificial intelligence	2020-01-01	2020-06-30
EXIMIOUS	Mapping Exposure-Induced Immune Effects: Connecting the Exposome and the Immunome	2020-01-01	2024-12-31
SPRING	Socially Pertinent Robots in Gerontological Healthcare	2020-01-01	2023-12-31
MOOD	Monitoring Outbreak events for Disease surveillance in a data science context	2020-01-01	2023-12-31
XpeFundus	A smart hyperspectral system for early, non-invasive diagnosis of retinal disease	2020-01-01	2020-04-30
PerMedCoE	HPC/Exascale Centre of Excellence in Personalised Medicine - PerMedCoE	2020-10-01	2023-09-30

Source: CORDIS, https://cordis.europa.eu/en, retrieved on 15.03.2021.

### 1.1 Value

It is the method of extracting useful information from huge datasets [4]. The process is based on how the data is added to creating knowledge [5]. “It is well and good having access to the data but unless we can turn into value it is become useless”. Therefore, it is the most important feature of big data, in which several institutions and enterprises invest the most in order to generate knowledge and incomes [6].

### 1.2 Variability

It refers to the consistency of the data over time [7].

### 1.3 Variety

It represents all type of information coming from structure data, semistructure data and unstructured data: relational tables, key-value web clicks, email messages, articles, streamed videos and audios, among others [4, 5]. This information is gathered via sensors, smartphones or social networks [5]. In healthcare realms, the information comes from both structure and unstructured sources: omics, electronics health records (EHR’s), clinical trials results, physiological and chemical sensors, among others [8].

### 1.4 Volume

It refers to large amounts of any kind of data currently available from any different source and its management [4]. The length of the data goes from Terabytes (TB: Approximately 10^12^ bytes) to Zettabytes (ZB: Approximately 10^21^ bytes) [5]. An example in the biomedical field is Proteomics DB, a proteome database created by the University of Munich, which gather a data volume of 5.17 TB, containing 92% of the known human genes. In medical imaging, for instance, the Visible Human Project archived 1871 male colour cryosections and 5189 female anatomical images, around 32 MB and 40 GB, respectively [8].

### 1.5 Velocity

It is the speed of data generation, data transfers, data collection and data processing [4–6]. The data content is constantly changing due to the absorption of complementary data collections, addition of new data and the different forms of streamed data from multiple sources with different speeds [4]. Nowadays, sequencing technologies enables the production of billions of DNA sequence data at a relatively low cost, the information obtained is stored in desk computers and shared within institutions worldwide [8].

### 1.6 Veracity

It is the reliability and accurateness of information, as well as the data quality, correctness of the data and data governance [4, 5]. This attribute depends entirely on the data source [6].

## 2 Architecture of big data

**Figure 2:**
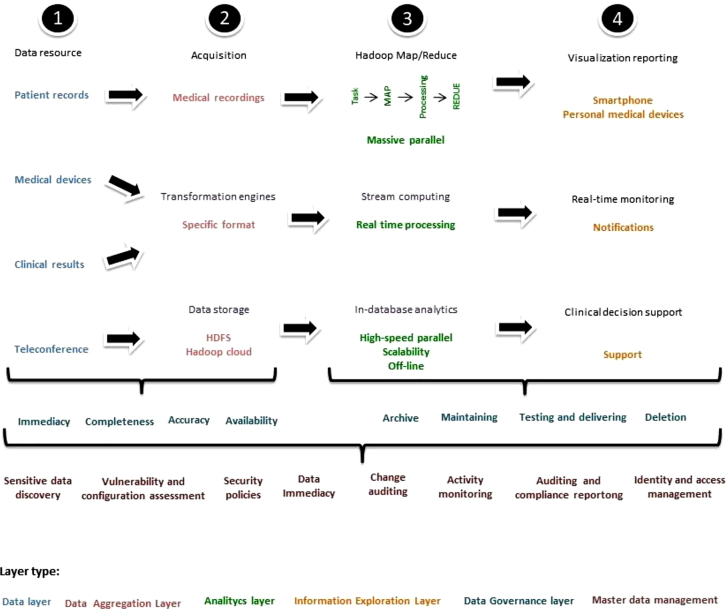
Big data analytics architecture in healthcare. The architecture of big data rule by the capture, transformation consumption and data management regulation. The first process consists of the recollection of structured, semi-structured and unstructured information from different devices. Then, the information is converted in specific formats and stored. Later, the data is processed by map/reduce other programming models which analyze different features of the data according to the needs of the user; early outputs are generated. Finally, more convoluted outputs are created and delivered to electronic devices. All the processes are based on data security statutes, master data management and data life-cycle management, ensuring the good quality and the correct use of the information.

**Figure 3:**
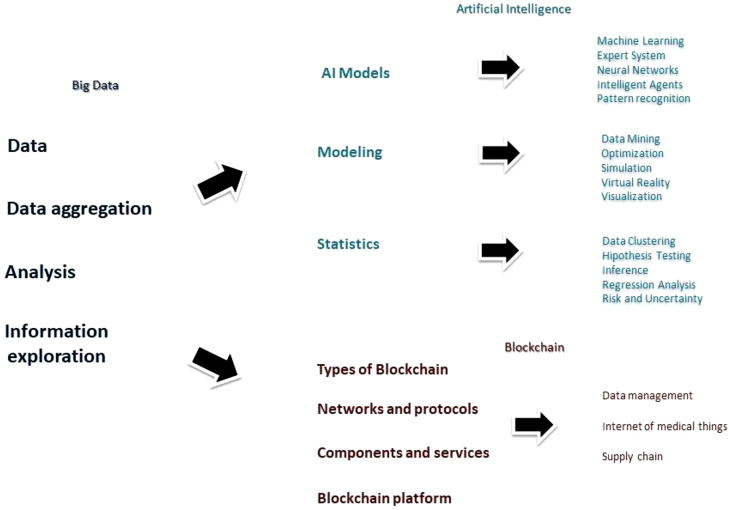
Big data, artificial intelligence and blockchain in healthcare. The three technologies have a great synergy. Big data acts as the main source of information of both AI and blockchain, not only for the data recollection but also for the analytic and management processes. The early process of the information with big data allows both technologies to acquire refined data. IA and blockchain amplify and spread the second use of the information, elaborating more convoluted analysis of the information and producing advanced outputs.

Three works [9–11] suggest a Big Data analytics architecture in healthcare, which exemplify how the data is collected, transformed and consumed (Figures 2 and 3).

### 2.1 Data layer

This layer contains all the data needed to support healthcare activities. The data is obtained and deposited in databases from internal and external sources [9]. The data comes from EHRs, electronic devices, back-end data of mobile healthcare Apps and streaming data generated by wearable medical sensors. Additionally, the data is pre-processed to be ready for the next stages [10].

### 2.2 Data aggregation layer

The data should be collected, transformed, and processed at this level. Firstly, the data was collected from different sources [9]. A transformation engine then pushes, filters, cleans, separates, translates, merges, sorts and validates the data [9–11]. Ultimately, for further processing and analysis, data is loaded into target relational databases such as the Hadoop Distributed File Systems (HDFS). The concepts of data management are ruled by legislation covering enforcement, data governance policies and access controls. Methods for data storage can be implemented and completed in batch processes or in real time [9].

### 2.3 Analytics layer

The analytics layer, where data processing takes place, is based on basic statistical analysis [10]. It is divided into three major components: Hadoop Map/Reduce, stream computing and in-database analytics. It promotes evidence-based medical practices and enhances preventive healthcare, clinical and surgical management by examining electronic health records (EHRs), treatment trends, quality of care and patient behaviours [9].

### 2.4 Information exploration layer

It consists in output visualization, real-time monitoring, and clinical insights and decision-making support [10]. This level is important for Big Data analytics, as the data supports the everyday activities of users and lets managers make quicker and better choices [9]. Currently, some artificial intelligent algorithms such as Bayesian model, logistic regression model, decision tree, support vector machine and random forest can be integrated with domain knowledge for clinical decision purposes [10]. In addition, real-time information tracking allows users to monitor the patient current health status, enabling physicians to have a swift intervention when it is necessary. This information can be sent in real time to interested users and made available as reminders and proactive alerts [11, 12].

### 2.5 Data governance layer

Three modules are contained in this layer: (I) The master data management module assures the immediacy, completeness, accuracy and availability of master data by standardizing, removing and integrating data. (II) The data life cycle management module is a continuous method for keeping the data accurateness over time. It is able to archive data, manage data warehouse, test and deliver various application systems, and remove and dispose information. (III) The data security and privacy management module is the framework for testing and validating the configuration, as well as publishing, auditing, and master data protection [9].

## 3 Tools and techniques of big data: Hadoop and map/reduce

Hadoop is an open source tools, libraries and methodologies for big data analysis in which a number of datasets are collected from different sources [12]. It was inspired in the project of Google File System (GFS) and the MapReduce programming paradigm [13].

Two main modules compose Hadoop:

(1) Hadoop Distributed File System (HDFS): Clustered data is broken in small pieces called “blocks or data nodes”, in which the actual data information is saved in proper sequence, having equal in size among blocks [12, 13]. Other node called the single name node manages all the metadata needed to store and retrieve the current data from the data blocks. Afterwards, Map and Reduce functions can be implemented in small subsets, offering the scalability required to process huge volumes. Each block is stored several times, and to ensure consistency, at least one block is housed in separate racks [13].
(2) Hadoop MapReduce: The programming model implemented by MapReduce is based on mappers (Map) and reducers (Reduce). It is counted as a processing area. Map designs map processors or work nodes, which produce a set of intermediate key/value pairs (Tuples). Reduce acquire the output from Map and combine all the Tuples that are associated with the same intermediate key in smaller subsets to get the last outcomes. An intermediate stage is known as Combine/Shuffle and it obtains the Tuples from Map and decides which node will process the data, leading the output of one task from Reduce [4, 14].

## 4 State-of art of big data in healthcare

In recent years, the use of big data has increased in healthcare realms due to the need for many fields to automate diagnosis, improve the identification of illness, establish rapidly medical treatment, predict patient outcomes and avoid catastrophic events [15]. The current use of Big data analytics in clinical and scientific fields is classified in three categories: (I) data storage and retrieval, (II) data security and (III) data analysis [16].

## 5 Data storage and retrieval

HDFS is used mainly for storing EHR and other clinical studies [16]. Moreover, HDFS works with real-time stream data and monitors patient vital signs as a result of the conversion of a huge amount of unstructured data generated by sensors that track patient health status, such as heartbeats per minute, blood pressure and respiratory rate. When any issue occurs, an alarm will be produced automatically and sent to the medical staff to ensure patient safety [14, 17].

For instances, a novel platform called Cloudwave was created to store and share within clinical institutions EKG from patients with epilepsy. It facilitates real-time interaction with large quantities of electrophysiological signals and features a web-based interface that uses signal analysis and querying functionalities. The main objective is to ease the second use of the data and to identify risk factors for sudden death in epileptic patients using EKG parameters [18].

### 5.1 Data security

One downside about Big data lies on the data retrieval and how this action exerts greater pressure on data security. Therefore, there are mechanisms to assure that the information is protected, shielded and well used. One example is a Lucene based distributed search cluster called Home-diagnosis, which is a cloud-based platform focus in self-caring services, supporting medical record retrieval, data analysis and strong privacy protection. This platform allows patients to get diagnosis by themselves with similar patients’ records and clinical studies but having a special care with the identity of the donor data [19].

### 5.2 Data analysis

Forecasting the risk of disease and progression over time can be very useful for promoting clinical decision-making, diminishing morbidity and mortality rate, decreasing use of medical supplies and preserving a good quality of life for patients. Several clinical fields are leveraging the use of big data analytics, and cardiology is on of them. A MapReduce prototype was made to diagnose mild hypertrophic cardiomyopathy in young athletes faster and with more accuracy. The algorithm contains around 10,000 medical records and is based on extensive tests against large medical datasets to identify potential correlations and assess the overall algorithmic quality and diagnostic accuracy. The final results of the first stage were fruitful; (1) the predictive accuracy was optimized and (2) the time analysis was decrease from 9 h to only a few minutes [20].

## 6 Big data and artificial intelligence in healthcare

Artificial intelligence (AI) refers to a system or process responding to an input from the environment, and then adjusting its operation to enhance an index of outputs [21]. The recent emergence of AI in healthcare can be attributable to two factors: (a) general purpose accessibility and open source AI frameworks that enable non-AI practitioners to apply complex AI algorithms without the need for advanced AI training, and (b) the ability to incorporate heterogeneous information to create broad datasets to investigate complex health management problems [22].

Since Big data analytics allow us to accumulate all knowledge, there could be a synergy with AI on the behalf of the medical realm [21]. Far beyond hospitals, these new analytical methods allow “deep learning health systems” to move science, education and even treatment wherever it is need it [23].

There are five types of analytics made by Big data and AI:

(1) Decision analytics helps to assist healthcare professionals by providing tailored diagnostic and treatment choices, identifying the most appropriate and evidence-based treatments, creating care plans, improving warnings for possible adverse effects, and stratifying patients with respect to illness risk, therapy response and health outcomes [22].
  Despite the large datasets and the complexity to analyse MRI lesions, both technologies are used to identify vascular and parenchymal lesions in MRI studies automatically in patients with chronic stroke. The project called Lesion Identification with Neighbourhoods Data Analysis or LINDA, is an algorithm capable of learning the relationship between current manual segmentations and a single T1-weighted MRI. A database with 60 left-hemispheric chronic stroke patients was created and tested against an independent dataset with 45 patients. The aim of the project was to measure the accuracy and the inter-observer reproducibility of the technology by means of the estimated predicted manual lesion and predicted lesion volume. The results were promising: (1) the correlation of both variables in the single evaluation was *r* = 0.961, meanwhile the correlation when evaluating the two databases was *r* = 0.957 [24].

(2) Predictive analytics involves the creation of data-driven models based on a collection of multivariate predictor hallmarks. The models learn the historical trend within a dataset and then use the learned trend to force a future value, outcome or event given input values of the predictor hallmarks. Thus, it can forecast the evolution of a patient, the success of a pharmacological or surgical treatment or the incidence of a determined illness [22].
  Infection outbreaks are so relevant nowadays due to the effect caused in the social, economic and health spheres. The prediction of catastrophically events such as outbreaks may diminish the spread of the microorganism; reduce the number of cases, mortality and morbidity rates. A currently example is the Canadian firm known as Blue Dot, which designs a software to predict the next COVID 19 outbreak position through Big Data and AI. This was the first company who announce news of the outbreak in late December. Currently, this enterprise is working for the Canadian government and other private firms as a consultant [25].

(3) Prescriptive analytics is an action suggestion activity that simulates outcomes for all possible scenarios. Therefore, it demands a complete, comprehensive and precise knowledge about the process being simulated. This analytic branch is on early stages due to its high level of complexity to create and validate sideways scenarios for convoluted medical conditions [22].
(4) Comparative analytics creates an objective and concise probabilistic model of comparison based on a combination of associated characteristics between two entities or outcomes. The aforementioned model reflects an induced causal relationship between a set of attributes leading to a specific result. Those models are used to compare pathology test ordering trends of healthcare providers treating a specific case mix of patients, hospital readmission costs across different specialties and diagnostic testing impact on medical practices [22].
(5) Semantic analytics is a knowledge-driven methodology that recognizes and leverages the implicit semantic relationships between the attributes of the data to discover new information and address specific and complex interrogatives [26]. Semantic analytics uses a web platform to incorporate heterogeneous data using linked data concepts to extend the scope of query answering data and infer plausible answers by logical reasoning and creating new and logical semantic connections among the known concepts. It is an excellent tool for analysing biomedical, genomic and drug datasets to pinpoint previously unknown interactions between drugs, drug side effects, genotype-phenotype influence, pharmacological outcomes and gene-drug response [22].
  On the other hand, this technology can also support decision-making when the data from EHR and other sources is incomplete. As an example, a multi-strategy reasoning method with an integrative deductive and logical reasoning was developed in order to shrink data gaps and generate information based on the available clinical findings [26].

## 7 Blockchain and machine learning in healthcare

Machine Learning is a sub-area of artificial intelligence. The term refers to the ability of intelligence systems to recognize patterns in databases with statistical and mathematical methods and learning from mistakes based on experience on existing algorithms to finally develop solutions. Blockchain technology makes sure that data is decentralized legitimate and secured, with the key difference between a typical database and blockchain being the way the data is structured. A database structures its data into tables whereas a blockchain structures its data into blocks that are chained together. Convergence of these two technologies can give highly accurate results in terms of machine learning with the security and reliability of Blockchain Technology, as Chen et al. showed with their work named “LearningChain” [27] and most recently Ziyuant et al. with their framework “CrowdSFL” [28]. Examples where machine learning and blockchain intertwined, include (1) CloudBurst [29]; (2) ExplorerChain [30].

### 7.1 CloudBurst

CloudBurst is a computing paradigm that aids in the process of genome mapping, improving the scalability of reading broad sequencing data, it parallelizes the short-read mapping process, increasing speed to process 24 times faster than computing models.^[Schatz 29]^

### 7.2 ExplorerChain

ExplorerChain combines artificial intelligence and blockchain technologies using decentralized networks in healthcare/genomic datasets to provide several enhancements, such as regulation of the use of computational resources, immutable audit trail, single point failure avoidance, security improvement, among other. It was showed that ExplorerChain had similar discrimination power, but slightly increased running time in comparison to central server-based algorithm [30].

## 8 Big data and blockchain technologies in healthcare

Blockchain is a public ledger responsible for conducting transactions and store any committed activity in a block list. This chain grows as new blocks are continually appended to it. Asymmetric cryptography and algorithms with mutual consensus are introduced for user protection and accuracy of the ledger. In general, the Blockchain technology can save cost considerably and increase performance due to its core features of decentralization, durability, transparency and auditability [31]. In 2009, the use of Blockchain technology was introduced for the first time in Bitcoin transactions. Bitcoin was the first cryptocurrency in history, and among others, the most important. Cryptocurrencies are similar to fiat money such as USD or EUR, enabling value exchange by using cryptographic protocols as the basis for governance rather than as a central authority mechanism such as banks, leading Bitcoin network transactions occur without any third party [32]. Blockchain and Big data are used in many ways in healthcare, such as data management, supply chain management and Internet of medical things [33].

### 8.1 Electronic health records

Electronic health records offer a dynamic network that collects and handles data from patients and monitors how data is managed. Thus, the data can be easily used within the hospital or can be shared between institutions in order to provide an integrative approach and reliable choice of care, maintaining data confidentiality and proper use of the information [34]. A clear example is Medshare, a system that offers a blockchain-based exchange of electronic medical information between data custodians and trustless parties. It integrates data provenance, auditing, monitoring of shared medical data and trailing of activities. By means of smart contracts and access management schemes, they can monitor user activity effectively and revoke access and permissions to violated user [35].

### 8.2 Remote patient monitoring (RPM)

It includes collecting biomedical data through body area sensors and mobile devices to allow remote monitoring of patient condition outside healthcare environments. Blockchain and Big data were introduced as a medium for the storage, exchange and retrieval of biomedical data collected remotely [36]. The outcome data can also be shared with healthcare stakeholders through mobile devices [36]. In particular, a mobile-enabled diabetes tracking system named SMEAD tracks various parameters through wearables, monitors and forecasts the patient status and alerts patients of the intake of the drug. The dose is monitored in patients needing insulin, whilst the insulin itself is maintained under adequate cooling conditions. A warning is sent to the caretakers through social networks in case of an emergency such as skipping a dose or elevated blood sugar levels [37].

### 8.3 Genomics

Some firms are developing technology with Big data and Blockchain to enhance and accelerate experimental processes. Furthermore, they can exchange knowledge openly or at a premium, monetizing and gaining an extra profit to invest in additional studies [38].

### 8.4 Pharmaceutical supply chain management and pharmaceutics

Approximately 60% of pharmaceutical companies work with Big data and blockchain to automate drug production, patent security, pharmacy surveillance and drug distribution processes by ensuring that all events are registered in tamper-proof blockchain nodes, and using smart contracts that provide credibility, traceability and accountability [34, 36].

Some companies are spending exhaustively in applications that detect prescription drug fraud. The goal is to record any transaction related to the prescription of drugs on the blockchain network to which all stakeholders are linked and to detect any alteration or malicious manipulation of the prescription [39].

### 8.5 Clinical trials management

Big data and Blockchain allow for an effective and trusted method to accelerate the clinical trials and testing process, following each step-in detail. Furthermore, they enable the auditors to provide a convenient and clear mechanism for legal and ethical validations of the trial process. At the same time, they manipulate the consent and the real time data, and evaluate the outcome in a trusting and transparent way [40].

Also, they can provide models for exchanging data from clinical trials that enable for transparency and reproducibility through smart contracts. Those smart contracts can also handle the data in multi-site clinical trials, reducing the administrative burden, time and effort to ensure data integrity and privacy [34].

### 8.6 Claims and Billing Management

Because of their transparency, decentralization, immutability and auditability of information stored therein, Blockchain and Big data can benefit the processing of health insurance claims in healthcare [35]. Both technologies bring together patients, providers, and insurers into one network to provide real-time insights about the healthcare experience of patients, easing the management of insurance claims [38].

## 9 Benefits of Big data in healthcare

Healthcare data is plentiful, but it is stored and withheld in facilities, clinics, hospitals or insurance companies, resulting in resource underuse, redundancy and inefficiency. However, stakeholders are rising their voices and they demand for an enhancement in the exploration and exploitation of the existent data [40]. Using Big Data, healthcare organizations could build networks to make substantial changes in the medical education, clinical practices and research. It supports the storage, sorting and analysis of patient information, and enhances the identification and prevention of diseases, therapy assessment, surgical planning and outcome prediction [41]. In combination of computer science, clinical semiology, radiology, advanced imaging, genomics and biochemistry, Big data drives a promising development of a wider range of technical tools, pharmacological therapies, surgical approaches, among others [42].

### 9.1 Economic benefits

Although the highest developed countries spend a large amount of gross domestic product on health, these expenditures do not tend to boost health outcomes. Additionally, the increasing cost of medical care and health benefits suggests that more strategies should be implemented to improve the healthcare systems’ effectiveness and to save on public investments [43]. As reported by the US, it has not increased its life expectancy or last-days quality of life, even having a one of the highest healthcare expenditures of any developed country [44]. The rashly aging of the population and the raising of chronic diseases lead an increment of economic demands. It is estimated that by 2020 the number of people aged 85 and older will increase from 14 million to 19 million, and by 2050 to 40 million. The impact of the ever-increasing demands is clearly seen in European countries where operational losses have been registered by a third of their hospitals [45].

By implementing Big data as a tool for forecasting models, it can enhance the economic aspects of healthcare for developing expenditures projections in potential healthcare hazards, explore different policy scenarios that solves the convoluted circumstances, and apply the most effective strategy against the threat. It is expected that Big data might generate more than $300 billion in savings per year in U.S. healthcare [44].

### 9.2 Technological benefits

Portable devices and social media have become the center of data collection and outcome feedback due to the closeness, portability and familiarity with the population. In healthcare scenarios, the EHR and physiological sensors are considered as the key points in patient follow-up and monitoring. All those devices generate a movement towards the renovation of data resources. Thus, the management and analysis of the information should also evolve, and Big data might make this transition come true. For instance, Big data on smartphones creates an efficient tool for transmitting motivational and medical messages to patients in order to engage them in lifestyle enhancements and to achieve an excellent treatment attachment, improving their health and wellbeing [46].

### 9.3 Other benefits

According to Wang et al. [9], Big-data analytics related benefits can be divided into five groups: Information technology (IT) infrastructure benefits, operational benefits, organizational benefits, managerial benefits and strategic benefits. The two most convincing advantages are IT infrastructure and operational benefits due to the current Big data analytics performance. In the first category, Big data showed that it can reduce system redundancy, avoid unnecessary IT costs, transfer rapidly data between healthcare IT systems, use properly the healthcare systems, standardize processes among healthcare IT systems, and reduce IT data storage costs. For operational benefits, Big data can improve the quality and accuracy of clinical decisions, process a large number of health records in seconds, reduce the time of patient travel, access immediately to clinical data to analyze, shorten the time of diagnostic test, reduce surgery-related hospitalizations and explore inconceivable new research avenues. Contrarily, Big data is at an early stage for organizational, administrative and strategic advantages, and the progress at those levels requires more time and technology. However, in the organizational heading, it seems that Big data may detect interoperability problems much more quickly, improve cross-functional communication and collaboration among stakeholders, allow to share data with other institutions and add new services, content sources and research partners. At managerial level, Big data might acquire acuity about changing healthcare trends in the market, support decision-making to members of the board and heads of medical departments on the daily clinical setting and optimize of business growth-related decisions. On the strategic side, Big data might provide a big picture view of treatment delivery for meeting future needs and create highly competitive healthcare services.

## 10 Limitations of big data

The main limitations of Big data are found when data quality is compromised and when users fail to use correctly Big data analytics.

### 10.1 Data limitations

Some of the limitations of Big data revolve around data and its quality. As mentioned before, the source of the data is responsible for this. However, data storage and management also play a role in data features. Data adequacy, data accuracy, data completeness and the nature of the reporting source are the most important features about data quality. Their aberrant modifications are threats for users when using Big data that jeopardize the veracity of the analysis results [47]. Therefore, Big data is prone to have erroneous outcomes due to the corruption of the data quality, generating wrong assumption on users. Those wrong assumptions may create false knowledge with a huge margin of error. Not only the data quality, but also the sample size and data selection matter. Sometimes, the datasets are not big enough compared with the number of attributes that allow statistically significant analysis. On the other hand, the data is abundant, but users choose arbitrarily the information, forgetting about the representative of the data as a whole, thus, they commit a selection bias. In both scenarios, the acuity of Big data analytics and their results may be compromised [47].

### 10.2 Big data limitations per se

Users are primarily involved in those limitations. Big data is a tool, which stakeholders use in different scenarios. Nonetheless, it is important to know when Big data could be the option to solve certain problems. So, users should know the characteristics of the data and observe the compatibility among datasets in order to achieve a coherent analysis. “It is difficult to compare the numbers of apples with numbers of oranges” [48].

To use correctly Big data analytics, one big step is to set the main goal or end product of the application. Second, users should learn as much as possible about and understand the possible mechanism behind the studied object or phenomenon. Third, they should hypothesize the possible outcomes. Finally, they should compare the final result with the main goal imposed at the first step. If users omit or make a mistake in at least one of the aforementioned steps, the Big data process will be wrong and the further precepts too [49].

## 11 Future of big data

### 11.1 Health education

Although great achievements were reached with Big data in research and healthcare areas, health education was developed to a lesser extent. Big Data may drive the learning process of manual skills according to the learner level, capture longitudinal data from educational institution to look for impact of changes in admissions policy, curricula and combine longitudinal and cross-sectional data of all learners in a region over a period of time, to create benchmarks for programs and schools, and to support a more detailed model of individual progress [49]. There are some expectations about the conjunction between Blockchain and Big data. Experts say that both technologies mainly may offer a platform for medical libraries manager and course certification. They might be in charge of the collection, storage and sharing of authoritative information by creating time-stamped, verifiable copies of journal articles. Furthermore, blockchain will protect the records, preventing any anomalous alterations. On the other hand, certification of medical courses may be provided to the right people without any assistance from third parties [34].

### 11.2 Big data and digital anatomy

Digital anatomy is the mixture of medicine, computer science, and information technology. It incorporates and reconstructs virtual structure images based on a vast amount of computed tomographic data of specimens of the human body, wall maps, models and images that fulfill the criteria of systematic anatomy, regional anatomy, and sectional anatomy. The synergy between big data and digital anatomy will enhance many healthcare areas. In the clinical realms, big data and digital anatomy may help fast disease prevention and diagnosis, quick and effective treatment establishment, and precise outcome prediction. In academic fields, anatomy courses focused on virtually-actual integrated and mutually-assisted teaching will minimize the use of large quantities of specimens, reduce costs, increase learning performance, arouse student learning interests and personalize education.

Storing anatomical information and adding up physiological components may create digital scenarios for research fields. It might be possible to establish simulations of scientific trials, test any kind of hypothesis, and obtain results and predicting human phenomena. The final goals are to facilitate scientific knowledge acquisition, reduce human and technological resources, and avoid dangerous exposures to any kind of living being.

## 12 Conclusions

Throughout the article, many applications of Big data alone and in conjunction with AI and Blockchain were shown in healthcare. Clinical grounds have gained several benefits, such as improving disease identification, developing rapid diagnosis, forecasting reliable outcomes, and concisely avoiding catastrophic events. Moreover, research has accomplished significant achievements, mainly in data recollection, data storage, data management and data analysis. However, there are unexplored areas in which the synergy of Big data, IA and Blockchain can be the answer. In a short to middle term, medical education may explode those technologies to facilitate information storage by creating platforms for information recovery, data sorting and data protection. Furthermore, the learning process might be tailored to the students according to their skills, their necessities and their performance.

Digital anatomy and Big data propose as the new technological bound that might solve unsettled healthcare enquires. It is expected that any healthcare provider activity might be enhanced, decision-making, science development and knowledge conveying. Conversely, all users should know about the limitations of Big data in order to avoid any erroneous action that infringe the data integrity or Big data correct activity. Any wrong step will compromise any Big data domain: Data recovery, data storage and data analysis.

The most relevant mistake of Big data application on healthcare is regarding decision-making and corrupted data analysis. Any decision based on imprecise information might provoke calamitous results, nevertheless, users feel a sense of “certainty” in the exact time point of making decision due to the legitimacy of the data provenance and its “flawless” process. It seems that it is better to have the data but not its second use than having a second use but wrongly processed. Having a false certainty is by far the worst thing anyone can perceive, because users are completely unable to recognize a mistake until it is too late. This problem might be caused by the use of any technology, and Big data is not the exception. Therefore, any process made by Big data should be evaluated time to time in detail in order to assure its accurateness. It can also renovate Big data by complement it with new technologies, innovating healthcare with unique data and knowledge, more efficient platforms and software, among other applications.

It is important to say that healthcare providers are not substituted by any machine or program. The advent of these technologies must be accepted as support for decision-making, error minimization, knowledge discovery and acquisition, and learning process.

Finally, current and further applications of Big data should follow ethical regulations and any user and stakeholder should comply them.
